# Kentucky Medical Society

**Published:** 1852-09

**Authors:** 


### KENTUCKY MEDICAL SOCIETY.

We are glad to see from the subjoined circular, that the *Committee of Arrangements* have directed public attention to the meeting of the State Medical Society soon to take place in this city, and we would cordially unite with the committee in the expression of a hope, that the meeting will be numerously attended. We have reason to know that great labor has been bestowed by some of the committees appointed to report on professional subjects at the approaching meeting, and cannot doubt that the meeting will be one of unusual interest.

—

The committee of Arrangements beg leave to call the attention of the physicians of the State to the second annual meeting of the Kentucky Medical Society, to be held in the city of Louisville in October. The meeting, according to adjournment, will take place at 1 o'clock, P. M., on the 20th of that month, in the Court House. The committee would inform their brethren throughout the State, that ample provision has been made for the reception and entertainment of the members, and hope that the meeting will be a large one, as it promises to be a highly interesting one. Various reports may be expected from committees appointed at the last meeting, and from the ability of the gentlemen engaged upon them, it is safe to promise that they will be full and instructive.

N. B. ANDERSON, M. D., *Chairman.*

